# Clinically Focused Computer-Aided Diagnosis for Breast Cancer Using SE and CBAM with Multi-Head Attention

**DOI:** 10.3390/tomography11120138

**Published:** 2025-12-10

**Authors:** Zeki Ogut, Mucahit Karaduman, Muhammed Yildirim

**Affiliations:** 1 Department of Surgery, Elazig Fethi Sekin City Hospital, Elazig 23300, Turkey; drzeki44@gmail.com; 2 Department of Software Engineering, Malatya Turgut Ozal University, Malatya 44210, Turkey; mucahit.karaduman@ozal.edu.tr; 3 Department of Computer Engineering, Malatya Turgut Ozal University, Malatya 44210, Turkey

**Keywords:** artificial intelligence, breast cancer, CNN, clinical decision support, computer-aided diagnosis, ViT

## Abstract

Background/Objectives: Breast cancer is one of the most common malignancies in women worldwide. Early diagnosis and accurate classification in breast cancer detection are among the most critical factors determining treatment success and patient survival. In this study, a deep learning-based model was developed that can classify benign, malignant, and normal breast tissues from ultrasound images with high accuracy and achieve better results than the methods commonly used in the literature. Methods: The proposed model was trained on a dataset of breast ultrasound images, and its classification performance was evaluated. The model is designed to effectively learn both local textural features and global contextual relationships by combining Squeeze-and-Excitation (SE) blocks, which emphasize channel-level feature importance, and Convolutional Block Attention Module (CBAM) attention mechanisms, which focus on spatial information, with the MHA structure. The model’s performance is compared with three commonly used convolutional neural networks (CNNs) and three Vision Transformer (ViT) architectures. Results: The developed model achieved an accuracy rate of 96.03% in experimental analyses, outperforming both the six compared models and similar studies in the literature. Additionally, the proposed model was tested on a second dataset consisting of histopathological images and achieved an average accuracy of 99.55%. The results demonstrate that the model can effectively learn meaningful spatial and contextual information from ultrasound data and distinguish different tissue types with high accuracy. Conclusions: This study demonstrates the potential of deep learning-based approaches in breast ultrasound-based computer-aided diagnostic systems, providing a reliable, fast, and accurate decision support tool for early diagnosis. The results obtained with the proposed model suggest that it can significantly contribute to patient management by improving diagnostic accuracy in clinical applications.

## Simple Summary

In this study, a deep learning-based model was developed that can classify benign, malignant, and normal breast tissues from ultrasound images with high accuracy, thus outperforming the methods commonly used in the literature. The model combines Squeeze-and-Excitation blocks, which emphasize channel-level feature importance, and Convolutional Block Attention Module attention mechanisms, which focus on spatial information, with the Multi-Head Attention structure to learn both local textural features and global contextual relationships effectively. In experimental analyses, the developed model achieved 96.03% accuracy on an ultrasound dataset and 99.55% accuracy on a second dataset of histopathological images.

## 1. Introduction

Breast cancer remains one of the most prevalent malignancies among women and continues to represent a major global health concern [1,2,3]. Many systems have been developed for the early diagnosis of this disease, which affects millions of people worldwide [4,5]. The main goal of these systems is to classify diseases accurately and implement appropriate treatment methods in a timely manner [5]. Accurately classifying early diagnosis is crucial for improving treatment outcomes, survival, and quality of life [2].

Breast ultrasonography (USG), an imaging method used in the diagnosis of breast diseases, has many advantages and disadvantages [6]. These include differences in radiologist assessments, difficulties in evaluating certain lesions, and individual dependency [7]. The limitations of this imaging method can affect the sensitivity and specificity of diagnosis [6]. Breast USG, which is known to be operator-dependent, has also been questioned for its diagnostic reliability and effectiveness in treatment planning [8].

At this point, the need for decision-support systems that can assist clinicians in guiding treatment is increasing [9]. Artificial intelligence, which has become a part of our lives in recent years, is being used increasingly effectively in medical diagnosis and treatment [10,11]. Owing to these methods, more objective diagnoses and higher accuracy rates have been achieved using systems developed from patient datasets [12]. Deep learning-based systems have yielded more effective results than machine-learning models in breast cancer screening and diagnosis [13]. Although many studies in the literature distinguish between benign and malignant lesions in breast diseases, normal breast tissue is not included in this classification. Failure to distinguish this distinction may lead to a defect in the diagnosis. Mixing these textures paves the way for wrong decisions to be made [14].

This study presents a new deep learning model designed to classify breast ultrasound images into normal, benign, and malignant breast tissues for the identification of breast lesions. The potential contribution of this model to the diagnosis and treatment of breast diseases in the clinic was evaluated. Precise identification and classification of lesion characteristics before surgical treatment will significantly contribute to the diagnosis and treatment planning. Higher lesion recognition reduces the number of negative biopsies and unnecessary surgeries. This artificial intelligence-based system aims to improve the quality of treatment and the early and accurate diagnosis of patients with suspected breast cancer.

There are studies in the literature on the prevalence of breast cancer in women. Rahman et al. stated in their study that breast cancer is the most common invasive cancer in women. They also noted that breast cancer has the second-highest mortality rate worldwide. The study aimed to detect breast cancer using architectures such as U-Net and YOLO. In the study, the researchers performed a data multiplexing step after the preprocessing step. An accuracy rate of 93% was achieved in the study [15]. Shah et al. stated that breast cancer is one of the most dangerous and common cancer types. Therefore, the importance of detecting breast cancer using artificial intelligence-based methods was emphasized. The study favored the use of CNN architectures. In this study, an ensemble deep learning model was developed combining the outputs of EfficientNet, AlexNet, ResNet, and DenseNet. The model achieved an accuracy of 94.6% [16]. Kormpos et al. used transfer learning-based methods for automatic breast cancer detection. In this study, conducted on ultrasound images, results were obtained with different models. Different results were obtained from different architectures in the study. However, the highest accuracy achieved in the study was 93.1% with the NasNet-based architecture [17]. Wang et al. adopted a different approach, achieving high accuracy in classifying breast lesions using multimodal breast ultrasound data. In their study, the researchers reported that they used four different ultrasound images together for the first time and developed a reinforcement learning-based model for this purpose. The accuracy achieved in their study was 95.4% [18]. Rashid et al. used the ResNet architecture, a CNN-based architecture, for feature extraction. The features obtained with this architecture were optimized using Metaheuristic optimization algorithms, and the optimized feature maps were classified using an SVM classifier. The researchers achieved an accuracy of 94.4% with their proposed hybrid model [19].

The proposed SE-CBAM-MHA-based hybrid architecture offers high accuracy and clinical reliability in lesion classification of breast ultrasound images, thanks to the high discrimination provided by channel and spatial attention mechanisms, as well as the contextual awareness and interpretability advantages offered by global context information. The originality and contribution of the proposed model can be described as follows.

- The proposed model offers a multi-layer hybrid structure by combining CNN-based local feature extraction with Transformer-based global context modeling.
- Thanks to the SE and CBAM-based attention mechanism, which simultaneously calculates channel and spatial importance levels, small but critical morphological differences can be captured in low-contrast and heterogeneous ultrasound images.
- The integration of the Multi-Head Attention layer modeled long-range relationships between the lesion and surrounding tissues, ensuring the inclusion of contextual information in the classification decision.
- The use of Focal Loss in malignant classes with low sample sizes resulted in a significant increase in model sensitivity and F1 score in challenging examples.
- By providing unique methodological contributions to contextual relationship modeling, which classical CNN models lack, and by emphasizing channel and spatial importance, a new framework for breast ultrasound image analysis has been introduced to the literature.
- The developed model achieved 96.03% accuracy on the ultrasound image dataset and 99.55% accuracy on the histopathological image dataset. These values will make significant contributions to the literature in the classification of breast cancer ultrasound and histopathological images.

The second part of the article includes the materials and methods section, the third part presents the application results, and the fourth part presents the discussion section. The study concludes with a conclusion section.

## 2. Materials and Methods

### 2.1. Dataset

**Figure 1:**
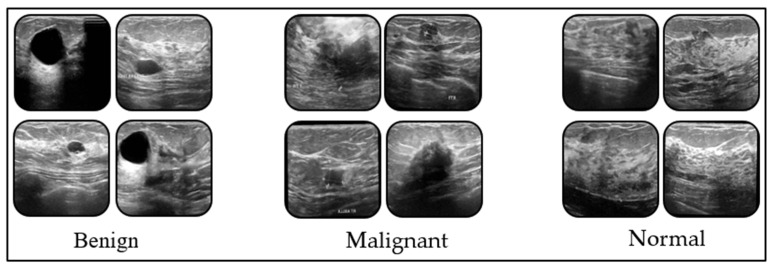
Dataset sample images for every classes.

The dataset used in this study was obtained from the Kaggle platform [14]. It was stated that 600 female patients were screened during the creation of the dataset. These women were between the ages of 25 and 60. The images are in .png format, and data augmentation was performed on classes with low data counts before training the models. The classes in the dataset are Benign (437 images), Malignant (420 images), and Normal (399 images). Sample images from the dataset are presented in Figure 1.

Benign: Most benign breast lesions do not carry a risk of malignancy, but some require periodic follow-up because they can exhibit radiological features similar to malignant tissue. Fibroadenoma, fibrocystic changes, cystic structures, non-neoplastic changes, lipomatous and papillomatous structures are included in this group. Excision may be considered for suspicious lesions in patients with suspected increased risk of malignancy and lesions requiring follow-up.

Malignant: Most breast malignancies are the histopathological subtypes of invasive ductal carcinoma and invasive lobular carcinoma. On breast ultrasound, these lesions may exhibit findings such as irregular borders, hypoechoic heterogeneous echoes, acoustic shadowing, spiculated contours, and increased blood flow. Early diagnosis and accurate treatment planning are crucial for this group.

Normal: Breast imaging of patients in this group reveals normal glandular and fibro glandular parenchyma along with fatty tissue. Breast ultrasound reveals homogeneous and evenly distributed parenchymal tissue. Since this group does not have any pathological features, there is no need to follow up with Breast USG.

### 2.2. Proposed Model and Other Models Used in the Study

**Figure 2:**
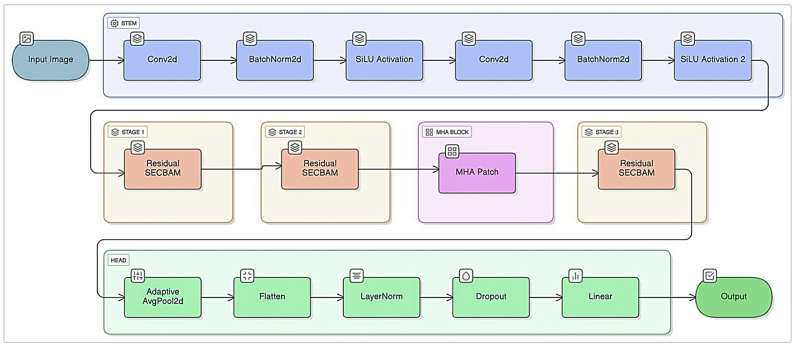
Proposed method.

The SE-CBAM-MHA-based model developed in this study aims to automatically classify breast tissues obtained from ultrasound images into three different classes: benign, malignant, and normal. The resulting structure of the proposed model is shown in Figure 2.

The architecture of the proposed model is designed as a hybrid structure consisting of four primary components. These components include convolutional feature extraction layers, SE-CBAM blocks containing channel and spatial attention mechanisms, a multi-header attention (MHA) layer inspired by the Transformer architecture, and fully connected layers used for final classification. This structure significantly improves classification performance by capturing both local texture patterns and large-scale contextual relationships.

The normalization process, initially applied on a pixel-by-pixel basis, is given in Equation (1).

(1) $$I_{norm}\left(x,y\right)=\frac{I\left(x,y\right)-\mu }{\sigma }\;$$

Here, $I\left(x,y\right)$ represents the original pixel value, and $I_{norm}\left(x,y\right)$ represents the normalized value.

The first convolutional layers of the model are used to extract low-level textural features. Equation (2) is used to calculate the convolution process. The feature maps obtained in this layer support the learning of meaningful representations in deeper layers.

(2) $$F_{i,j}^{\left(k\right)}=\sum_{m=0}^{M-1}\sum_{n=0}^{N-1}I_{\left(i+m)(j+n\right)}W_{mn}^{\left(k\right)}+b^{\left(k\right)}$$

Here, I represents the pixel value, $F_{i,j}^{\left(k\right)}$k represents the output feature for the kth filter, $W^{\left(k\right)}$ represents the filter kernel and $b^{\left(k\right)}$ represents the bias term.

The Squeeze-and-Excitation (SE) block, a key component of the model, summarizes the information carried by each channel using a global average pooling process and learns channel importance weights based on this information. First, the global compression process is defined by Equation (3).

(3) $$z_{c}=\frac{1}{H\;.\;W}\sum_{i=1}^{H}\sum_{j=1}^{W}F_{c}(i,j)\;$$

Here, $z_{c}$ represents the scalar value obtained as a result of global average pooling of the c-th channel, H represents the height of the feature map, W represents the width of the feature map, and $F_{c}(i,j)$ represents the activation value of the c-th channel at position (i,j).

The channel excitation process is then performed using Equation (4). In this step, the channel representations obtained by global average pooling are rescaled by passing them through two fully connected layers. The channel size is reduced in the first fully connected layer, while the second layer expands it back to its original size. The Sigmoid Linear Unit (SiLU) activation function is chosen for the nonlinear transformation between the two layers. The SiLU function is defined as shown in Equation (5). SiLU provides a smoother activation compared to ReLU, allowing low-value inputs to carry some information, thus contributing to the preservation of fine textural details, especially in low-contrast ultrasound images. The sigmoid function applied at the output generates an importance coefficient for each channel by compressing the channel weights to the 0–1 range.

(4) $$s_{c}=\sigma \left(W_{2}\delta \left(W_{1}z\right)\right)\;$$

$s_{c}$ is the importance coefficient calculated for the c-th channel, $W_{1}$ is the weight matrix of the first fully connected layer, $W_{2}$ is the weight matrix of the second fully connected layer, δ is the activation function, and σ represents the sigmoid function.

(5) $$\delta \left(x\right)=x\cdot \;\sigma \left(x\right)=\frac{x}{1+e^{-x}}\;$$

$\delta \left(x\right)$ is the SiLU activation output, x is the input value and $\sigma \left(x\right)$ is the sigmoid function.

The rescaling process is defined by Equation (6). In this step, the contribution of each channel is dynamically adjusted by applying the channel importance coefficients obtained in the previous steps to the original feature maps.

(6) $$\hat{F_{c}}=s_{c}\cdot \;F_{c}\;$$

Here $\hat{F_{c}}$ represents the output feature of the rescaled c-th channel and $F_{c}$ represents the feature map of the original c-th channel.

Through this process, each channel is dynamically strengthened or weakened based on the information it carries. Activation of channels with high discrimination specific to lesions in images within each class is increased, while channels containing unimportant or noise are suppressed. Thus, the model creates a stronger representation by focusing on fewer, yet more meaningful features, thereby improving classification performance.

Following the SE block, integrated CBAM also implements the spatial attention mechanism along with channel-based attention. The features extracted through averaging and max pooling are combined, as given by Equation (7).

(7) $$M=\left[AvgPool\left(F\right);MaxPool\left(F\right)\right]\;$$

The combined feature map, with a channel size of *M,* yields the average pooling result using *AvgPool* (*F*), while *MaxPool* (*F*) yields the maximum pooling result.

This stage provides both the average representation and the maximum representation as input to the spatial attention mechanism. The goal here is to make the model sensitive not only to the average structure but also to dominant local differences. This enables the more effective capture of critical regions, such as lesion borders, in ultrasound images.

The second step of the spatial attention mechanism within CBAM, attention map generation, is calculated as shown in Equation (8).

(8) $$S=\sigma \left(f^{7\times 7}\left(M\right)\right)\;$$

*S* is the spatial attention map and $f^{7\times 7}$ is the 7 × 7 size of the convolution filter.

This stage determines which spatial regions in the image are more important. Clinically critical regions in ultrasound images, such as the irregular edges of malignant lesions, heterogeneous echo patterns, or micro calcification foci, receive higher importance coefficients through this attention map. Thus, the model focuses on diagnostically significant regions when making classification decisions, rather than focusing equally on the entire image.

The rescaled features are then calculated as expressed in Equation (9).

(9) $$F^{\prime }=F⊗S\;$$

Here, $F^{\prime }\;$represents the rescaled feature map with spatial attention applied, *F* represents the original feature map, and ⊗ represents the element-wise multiplication operation.

This process enables the more effective capture of distinctive anatomical features, such as microcalcifications, edge irregularities, or heterogeneous echo patterns surrounding the lesion. Thanks to CBAM, the model evaluates the location and structure of the lesion like that of a human observer, thereby making classification decisions more reliable.

A multi-headed attention (MHA) layer, inspired by the Transformer architecture, was integrated into the model to learn deep contextual relationships. While classical convolutional networks can successfully capture local features, they are insufficient in modeling long-range dependencies. The basic structure of the attention mechanism incorporated into the model is defined in Equation (10), where the input features are re-represented by three fundamental matrices. These fundamental matrices are defined as Query (Q), Key (K), and Value (V). Here, Q is the vector used by the model to query the relationship of a feature at a given location to other locations. K is a reference vector representing the features of each location and helps determine which locations are important for attention. V represents the information contained in the relevant location and is used to create the output by weighting it according to attention scores.

(10) $$Attention(Q,K,V)=softmax\left(\frac{QK^{T}}{\sqrt{d_{k}}}\;\right)V\;$$

Here, *Q* is the query matrix, *K* is the key matrix, *V* is the value matrix, and *Attention(Q, K, V)* is the resulting attention output.

Through this process, the model learns the relationship between each spatial location and other locations, and assigns weights to each feature based on its importance within the context. In terms of breast ultrasound images, this mechanism considers not only the local structure of the lesion but also its relationship with surrounding tissues.

The multi-head version is as given in Equation (11). Here, multiple query-key-value groups are calculated in parallel, and each head learns different relationships and feature subspaces.

(11) $$MHA\left(Q,K,V\right)=\left[{head}_{1};\ldots ;{head}_{h}\right]W^{O}$$

Here, *h* is the number of attention heads used, ${head}_{i\;}$is the output of the *i-th* attention head, and $W^{O}$ is the output projection matrix.

Thanks to this structure, the model can make more accurate predictions by evaluating not only local features but also contextual information across the entire image.

The extracted feature maps are reduced to a single vector using the global average pooling process. This process is given by Equation (12). At this stage, the high-dimensional feature map is reduced to a one-dimensional vector by averaging over the spatial dimensions of each channel. Thus, the model’s output is transformed into a more compact and meaningful representation for the classifier layer.

(12) $$g_{c}=\frac{1}{H\;.\;W}\sum_{i=1}^{H}\sum_{j=1}^{W}F_{c}(i,j)\;$$

Here, $g_{c}$ is the scalar value obtained as a result of global average pooling for the c-th channel, *H* represents the height of the feature map, *W* represents the width of the feature map, and $F_{c}(i,j)$ represents the activation value of the c-th channel at position (i,j).

After this stage, the classification process is carried out using the SoftMax function, as given in Equation (13).

(13) $$P\left(y=c|x\right)=\frac{e^{z_{c}}}{\sum_{k=1}^{C}e^{z_{k}}}\;$$

$P\left(y=c|x\right)$ is the probability that the input sample *x* belongs to the c-th class, *C* is the total number of classes, $z_{c}$ is the logit value of the c-th class obtained from the fully connected layer, and e is the base of the natural logarithm.

Using the Softmax function, the model’s output is converted into a normalized probability value between 0 and 1 for each class, with the sum of these probabilities equal to 1. This approach allows for the assessment of the model’s confidence level by grounding the decision-making process in classification problems on a probabilistic basis. The AdamW algorithm and cosine annealing learning rate planner were employed for model optimization, and the Focal Loss function was selected to mitigate class imbalance and the adverse impact of complex examples. The Focal Loss function is defined as in Equation (14).

(14) $$L=-\alpha {(1-p_{t})\;}^{\gamma \;}\log{(p}_{t})\;$$

*L* is the Focal Loss value, α is the class weighting parameter, $p_{t}$ is the estimated probability value for the correct class, γ is the focusing parameter and $\log{(p}_{t})$ is the logarithm of the probability of the correct class. Focal Loss provides a more effective error signal than the classic Cross-Entropy loss by directing learning from easy examples to complex examples, thereby avoiding time wastage. Throughout the training process, the model was evaluated using basic classification metrics, including accuracy, precision, sensitivity, and F1-score. A confusion matrix was also used to analyze performance differences between classes better.

To objectively evaluate the model’s performance compared to the proposed architecture, pre-trained deep learning models commonly used in the literature—ConvNeXt-Tiny [20], ViT-B/16, ViT-B/32 [21], ResNet50 [22], EfficientNet-B0 [23], and DenseNet121 [24]—were retrained and tested using the fine-tuning method on the relevant dataset. Classification results obtained from these models were analyzed in terms of basic performance metrics, including accuracy, precision, sensitivity, and F1-score, and a detailed comparison was conducted with the proposed method. This comparison aims to quantify the performance improvements and unique contributions of the developed model compared to existing deep learning approaches.

## 3. Experimental Results

**Figure 3:**
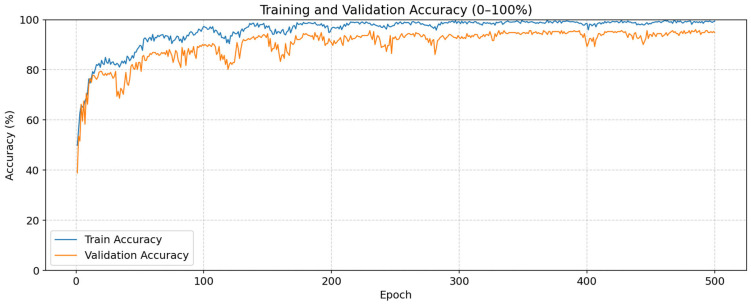

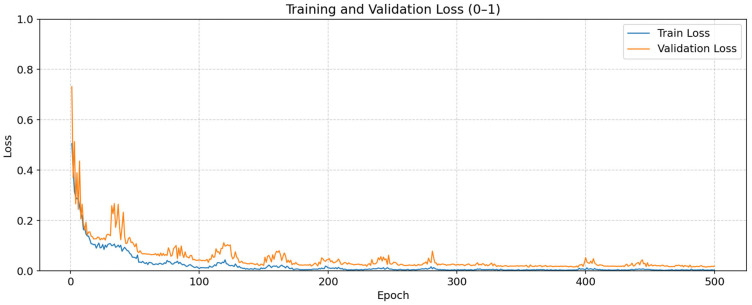
Accuracy and Loss Curves.

The results of this study, conducted for breast cancer detection, were obtained in the Python 3.12 environment. For use in the model, the images were first scaled to 224 × 224. AdamW was used as the optimization algorithm, and the learning rate was set to 3 × 10^−4^. The training and testing phases of the models were performed in the Google Colab environment using an NVIDIA T4 GPU. The results of the model developed for this process were compared with a total of six models accepted in the literature: three ViT-based and three CNN-based models. Accuracy, Precision, Recall, and F1-score metrics were used to evaluate the performance of the models. The data were split into 80% training and 20% testing sets for the proposed model and the models used for comparison. The proposed model was run for 500 epochs. The Training and Validation Accuracy lines and Training and Validation Loss curves obtained for the model developed for breast cancer detection are presented in Figure 3.

An examination of Figure 3 reveals that the model has a strong learning ability in terms of overall performance. The steady increase in accuracy rates throughout the training process indicates that the model effectively learned from the data. Furthermore, despite the high number of epochs, no significant drop in validation performance was observed, indicating that the model achieved stability. Training and validation losses, starting from high values at the beginning, decreased rapidly and dropped to very low levels from the 100th epoch onward. The performance evaluation metrics obtained in the proposed model are presented in Table 1 on a class basis.

When Table 1 is examined, it is observed that the highest F1-Score value of 96.43% was obtained in the Malignant class. To compare the performance of the proposed model, the performances of six different CNN and ViT architectures were also examined.

To compare the performance of the proposed model, results were obtained from the current CNN and ViT architectures. The performance metrics obtained for comparison are presented in Table 2. 

Model performance metrics were evaluated to 2 decimal places. When Table 2 is examined, it is seen that the highest accuracy value was obtained in the proposed model with 96.03%. The model that comes closest to the proposed model is DenseNet121 with an accuracy value of 94.84%. The lowest accuracy value was obtained in the ConvNeXt-Tiny model with 91.67%.

**Figure 4:**
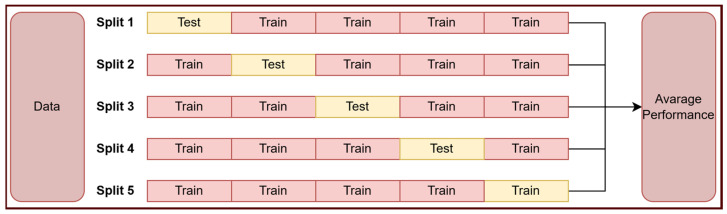
The 5-fold cross-validation process steps.

The performance of the proposed model was also tested on a second dataset. The second dataset consists of benign and malignant histopathological images. The Breast Cancer Dataset contains 5000 images per class [25]. Our proposed model was run on this dataset with 100 epochs. Our model used k-fold cross-validation on this dataset. K-fold cross-validation divides the dataset into equal parts and tests the model on a different fold each time. K-fold cross-validation increases the reliability of performance metrics. This also prevents the model from overfitting on limited datasets. At this stage, the k-fold value is selected as 5. The 5-fold cross-validation process steps are shown in Figure 4.

**Figure 5:**
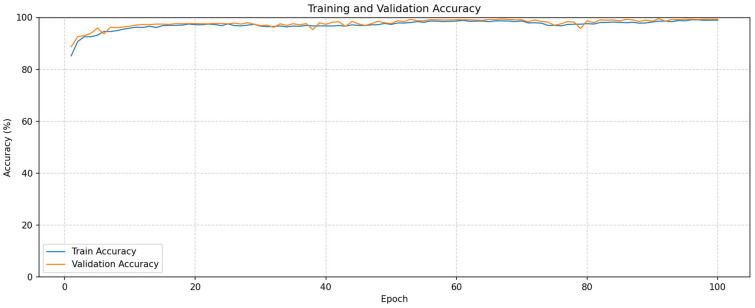
Best fold accuracy curves for training and validation of proposed model.

**Figure 6:**
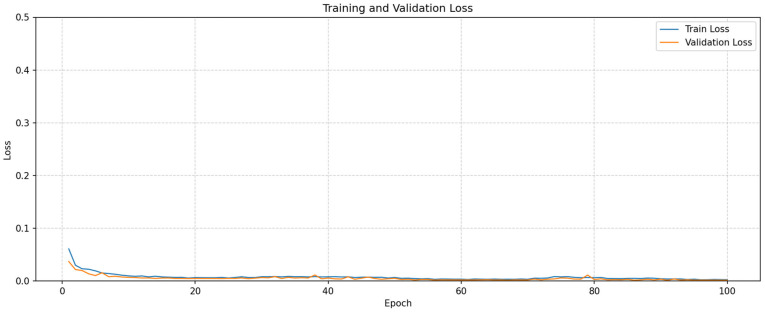
Best fold loss curves for training and validation of proposed model.

The training and validation accuracy graphs of the developed model are shown in Figure 5, and the loss graph is shown in Figure 6.

**Figure 7:**
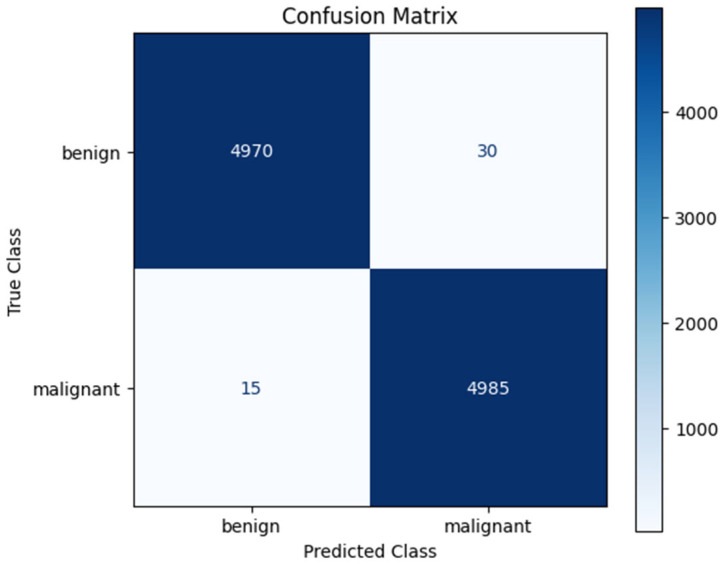
Confusion matrix of proposed model for second dataset.

Examining Figure 5 and Figure 6, the Accuracy curve is stable and high, while the Loss curve stabilizes at a similarly low loss rate. The confusion matrix obtained from our model trained via cross-validation is shown in Figure 7.

In the classification performed using histopathological images, our proposed model also achieved successful results on the second dataset. Examining the confusion matrix in Figure 7, it is seen that the proposed model correctly predicted 4970 of 5000 benign images. Similarly, the proposed model incorrectly predicted 15 of 5000 malignant images. The accuracy values for each fold are given in Table 3.

Table 3 shows the accuracy values for each fold after the cross-validation technique, followed by the mean accuracy value. The proposed model’s mean accuracy was 99.55%. Class-based performance metrics of the proposed model are presented in Table 4.

The average accuracy rate calculated at the end of cross-validation is 99.55, while the standard deviation value is ±0.05.

## 4. Discussion

Breast ultrasound is widely used because it does not expose the patient to radiation, is applicable even to patients at risk, and is superior to other imaging methods for evaluating dense breast parenchyma [26]. However, the variable results of breast ultrasound, depending on the operator, and its inadequacy in identifying some lesions are among its drawbacks [27]. The aim is to mitigate these negative aspects using clinical decision support suggestions provided by artificial intelligence-supported systems. This will contribute to more accurate decisions regarding diagnosis and treatment management [28].

High performance achieved in the model developed in our study will have many clinical benefits. Accurate rates for distinguishing between normal breast tissue, benign lesions, and malignant lesions will assist clinicians in follow-up decisions and medical or surgical treatment decisions. It offers many benefits, including reducing diagnostic biopsies, preventing unnecessary surgeries, diagnosing malignant patients before they reach advanced stages, and enabling early treatment planning. Many medicolegal situations can arise during the diagnosis and treatment processes of breast cancer. Artificial intelligence systems can be used in clinician decision-making processes and in multidisciplinary tumor boards where complex cases are evaluated [29]. Breast USG could be a new method for increasing the consistency between radiologists and surgeons regarding lesions that can be interpreted differently. The classification features of these models could be utilized when planning patients before surgical or medical treatment [30].

The use of artificial intelligence models for breast disease diagnosis has many benefits. In rural areas, where there are insufficient radiologists or clinicians, the availability of breast ultrasound contributes to early diagnosis, which is one of the most critical aspects of breast cancer. Artificial intelligence assisted imaging methods will help clinicians make accurate and early diagnoses in areas where breast specialists are scarce [31,32]. The use of datasets from densely populated urban areas in artificial intelligence models limits the generalizability of these data. Comprehensive datasets, along with the need to address ethical and data privacy issues, will further contribute to the development of artificial intelligence models for medical treatment [33]. The development of artificial intelligence modules for the diagnosis and treatment of breast diseases should incorporate changes in the diagnosis and treatment phases of patient groups of different ages and ethnicities. Datasets containing all clinical and radiological data used in the diagnosis phase will enhance the development and usability of these modules [34]. Such a transformation will lead to earlier diagnosis for many patients and treatment strategies that eliminate unnecessary methods. In this study, a hybrid model was developed for breast cancer detection. The developed model was tested on two datasets. Furthermore, the performance of the proposed model is compared with similar studies in the literature in Table 5.

An examination of Table 2, Table 3 and Table 5 reveals that the proposed model produces better results than similar methods and similar studies. Despite the high accuracy achieved in this study, the proposed hybrid model has some limitations. First, the model was trained on a public dataset consisting of only a single center and a limited number of breast ultrasound images. The histopathological image dataset used to test our model is also publicly available. The dataset’s single-center presence, inclusion of patients from the same population, and low number of rare cases in the dataset limit the generalizability of the study. Its applicability to demographics not included in this dataset should be questioned. The exclusion of demographic, clinical, and imaging data, as well as different imaging modalities, is a significant limitation in our study. Systems that combine demographic, clinical, and imaging modalities would make significant contributions to the diagnosis and treatment of breast cancer. Furthermore, although the image annotations in the dataset were based on expert opinions, labeling errors or class imbalances are potential factors that could affect model performance.

Future work aims to increase the generalization ability of the model by testing it on larger and more diverse datasets obtained from different institutions and devices. It is important to create a dataset by incorporating clinical and demographic information using metrics such as age, race, region, and blood values, taking into account factors that may cause the data to appear differently despite belonging to the same disease category. This will allow for more subjective model training and classification. Furthermore, by involving multiple centers, federated learning models will be developed to prevent data leakage between centers. With the increase in communication speeds between different regions and centers and the active adoption of 6G technology, future models aim to use quantum-based models for real-time classification, while the development of real-time artificial intelligence techniques is also considered if necessary during operation. Finally, developing methods to increase the explainability of the model is another goal.

## 5. Conclusions

In this study, a novel deep learning-based model was developed that integrates SE blocks, CBAM, and MHA mechanisms. The proposed model was developed to classify breast tissues as benign, malignant, or normal based on ultrasound images. The proposed model produced successful results by effectively capturing both local textural patterns and global contextual dependencies. The proposed model achieved a classification accuracy of 96.03%, outperforming similar studies in the literature, as well as commonly used CNN and ViT-based architectures, and state-of-the-art architectures. The obtained results highlight the potential of the developed model as a reliable decision support tool in breast cancer diagnosis. By significantly improving diagnostic accuracy and reducing human error, clinicians involved in breast disease management can achieve earlier and more reliable diagnoses. Furthermore, the proposed model can facilitate more precise treatment planning and has the potential to improve patient outcomes. Our future work will focus on expanding dataset diversity, incorporating multimodal imaging data, and validating the model in real-world clinical workflows to strengthen its applicability in clinical decision-making processes further.

## Figures and Tables

**Table 1:** Performance result.

Classes	Precision	Recall	F1-Score
Benign	98.80	93.18	95.91
Malignant	96.43	96.43	96.43
Normal	92.94	98.75	95.76

**Table 2:** Models performance comparison result (%).

Model	Accuracy	Precision	Recall	F1 Score
ConvNeXt-Tiny	91.67	92.00	91.77	91.71
ViT-B/16	92.46	92.60	92.59	92.53
ResNet50	92.86	93.06	92.94	92.91
ViT-B/32	93.25	93.37	93.38	93.29
EfficientNet-B0	93.65	93.73	93.78	93.70
DenseNet121	94.84	94.94	94.99	94.85
Proposed Model	96.03	96.15	96.03	96.03

**Table 3:** Accuracy rate of the proposed model per fold for the second dataset.

	Fold 1	Fold 2	Fold 3	Fold 4	Fold 5	Mean
Accuracy	99.48	99.72	99.61	99.58	99.42	99.55

**Table 4:** Cross validation performance metrics of the proposed model for the second dataset.

Classes	Precision	Recall	F1-Score	Number of Images
Benign	99.70	99.40	99.55	5000
Malignant	99.40	99.70	99.55	5000

**Table 5:** Literature Review.

Paper	Year	Methods	Performance
Rahman et al. [15]		U-Net and YOLO	Acc: 93%
Shah et al. [16]	2024	EfficientNet, AlexNet, ResNet and DenseNet based hybrid model	Acc: 94.6%
Kormpos et al. [17]	2025	NasNet	Acc: 93.1%
Wang et al. [18]	2020	Reinforcement learning-based model	Acc: 95.4%
Rashid et al. [19]	2024	CNN and Metaheuristic based hybrid model	Acc: 94.4%
Proposed Model	2025	SE-CBAM-MHA based hybrid model	Dataset1: Acc: 96.03% Dataset2: Acc. 99.55%

## Data Availability

A publicly available dataset was used in the article. https://www.kaggle.com/datasets/sabahesaraki/breast-ultrasound-images-dataset, accessed on 1 September 2025.
